# High-sensitivity C-reactive protein–triglyceride-glucose composite index and reduced estimated glomerular filtration rate in adults undergoing routine health examinations: a cross-sectional study

**DOI:** 10.3389/fendo.2026.1859383

**Published:** 2026-06-10

**Authors:** Hao Sun, Zhiqin Yu, Mengmeng Wang, Yuantao Qi

**Affiliations:** 1 Qingdao Municipal Hospital, University of Health and Rehabilitation Sciences, Qingdao, China; 2 Xiyuan Hospital, Chinese Academy of Traditional Chinese Medicine, Beijing, China; 3 Shandong Cancer Hospital and Institute, Shandong First Medical University and Shandong Academy of Medical Sciences, Jinan, China

**Keywords:** CTI, health examination, high-sensitivity C-reactive protein, metabolic inflammation, single-occasion reduced eGFR, TyG index

## Abstract

**Background:**

Reduced kidney function often develops in parallel with metabolic dysregulation and chronic low-grade inflammation, yet practical markers that capture both processes remain limited in routine screening settings. We evaluated whether the high-sensitivity C-reactive protein–triglyceride-glucose composite index (CTI), an integrated marker derived from hs-CRP and the triglyceride-glucose index, was associated with reduced estimated glomerular filtration rate (eGFR) in adults undergoing health examinations.

**Methods:**

This cross-sectional study included 1429 adults with complete core variables from an independent hospital-based cohort established at the Health Examination Center of the Second Affiliated Hospital of Shandong First Medical University between June 2024 and December 2025. CTI was calculated as TyG + 0.412 × ln [hs-CRP (mg/L)], where TyG = ln [TG (mg/dL) × fasting plasma glucose (mg/dL)/2]. The primary outcome was single-occasion reduced eGFR, defined as <90 mL/min/1.73 m², and was not interpreted as chronic kidney disease. Multivariable logistic regression, restricted cubic spline analysis, receiver operating characteristic analysis, subgroup analysis, variance inflation factor diagnostics, and sensitivity analyses were performed.

**Results:**

The mean (SD) age was 48.8 (11.3) years, 46.8% of participants were women, and 398 individuals (27.9%) had single-occasion reduced eGFR. The prevalence of reduced eGFR increased across CTI quartiles, from 12.8% in the lowest quartile to 45.7% in the highest quartile. After adjustment for demographic factors, lifestyle factors, cardiometabolic comorbidities, and uric acid, each 1-SD increment in CTI was associated with 45% higher odds of reduced eGFR (odds ratio [OR], 1.45; 95% CI, 1.11-1.90). Compared with the lowest quartile, the highest quartile showed an OR of 2.11 (95% CI, 1.07-4.17), whereas the adjusted linear trend across quartiles was borderline (P for trend = .056). Only 1 participant (0.1%) had eGFR <60 mL/min/1.73 m²; using a stricter eGFR <75 mL/min/1.73 m² threshold yielded a directionally similar but borderline association (OR, 1.75; 95% CI, 0.99-3.10). CTI discriminated reduced eGFR modestly but better than TyG, hs-CRP, and remnant cholesterol.

**Conclusions:**

In this health examination population, higher CTI was independently associated with a higher prevalence of single-occasion reduced eGFR. Because discrimination was modest and the design was cross-sectional, CTI should be interpreted as an adjunctive marker of metabolic-inflammatory burden rather than a diagnostic or predictive tool. Prospective validation is required.

## 1 Introduction

Chronic kidney disease (CKD) is now recognized as a major public health challenge because it is common, clinically silent in its early stages, and strongly linked to cardiovascular events, kidney failure, and premature death (1–3). Even before overt CKD is established, a downward shift in estimated glomerular filtration rate (eGFR) may indicate early reduction in renal filtration and a higher long-term cardiometabolic risk burden (3, 4). In the health examination setting, markers associated with early filtration reduction are of particular interest.

Renal functional decline rarely occurs in isolation. Increasing evidence suggests that early kidney impairment is embedded in a broader network of adiposity, metabolic disturbance, oxidative stress, endothelial dysfunction, and persistent low-grade inflammation (5–12). These interrelated processes also contribute to podocyte injury, altered intraglomerular hemodynamics, tubular stress, and progressive nephron loss (8–14). Accordingly, composite markers that reflect more than one pathogenic axis may be more informative than single biochemical parameters.

Insulin resistance is a central component of this network, but direct measurement by the hyperinsulinemic-euglycemic clamp is not feasible in large observational studies. The triglyceride-glucose (TyG) index has therefore been widely adopted as a practical surrogate of insulin resistance (15). Recent cohort studies, cross-sectional analyses, and meta-analyses have consistently linked higher TyG values to CKD, albuminuria, and future renal function decline (16–21).

Systemic inflammation provides another biologically plausible pathway to renal injury. High-sensitivity C-reactive protein (hs-CRP), a routinely available marker of low-grade inflammation, has been associated with reduced kidney function, CKD progression, and incident renal injury across several clinical settings (7, 22–25). Chronic inflammatory activation can worsen endothelial dysfunction, intensify oxidative stress, and amplify insulin-resistant states, thereby accelerating renal damage (7–12).

The high-sensitivity C-reactive protein–triglyceride-glucose composite index (CTI) was proposed to integrate these 2 dimensions into a single metric. Built from hs-CRP and TyG, CTI has recently been examined in relation to erectile dysfunction, coronary heart disease, cardiovascular disease, cardiometabolic multimorbidity, and CKD (26–32). Nevertheless, the incremental context of CTI in asymptomatic adults undergoing routine health examinations remains incompletely defined. In such populations, kidney-related abnormalities are frequently detected incidentally, and the relevant question is often whether a simple metabolic-inflammatory marker is associated with early filtration reduction or urinary abnormalities rather than with established CKD alone.

We therefore conducted a cross-sectional study in an independent hospital-based cohort of adults attending routine health examinations at the Second Affiliated Hospital of Shandong First Medical University. Our primary aim was to examine the association between CTI and single-occasion reduced eGFR (<90 mL/min/1.73 m²). To address clinical interpretability, we also described the number of participants with eGFR <60 mL/min/1.73 m², evaluated a stricter eGFR <75 mL/min/1.73 m² threshold, examined albuminuria and a combined reduced eGFR-or-albuminuria phenotype, assessed dose-response shape and discriminative performance, and performed subgroup, sensitivity, and multicollinearity analyses.

## 2 Methods

### 2.1 Study design and ethical approval

We established an independent hospital-based cohort consisting of individuals who underwent routine health examinations at the Health Examination Center of the Second Affiliated Hospital of Shandong First Medical University between June 2024 and December 2025. The protocol was approved by the institutional ethics committee (No. 2024R021), and all participants provided written informed consent.

Participants were eligible for the present analysis if they were adults and had complete data for the core exposure variables (hs-CRP, triglycerides, and fasting plasma glucose), serum creatinine for eGFR calculation, covariates used in multivariable models, and urinary renal phenotypes. The extracted analytic dataset contained 1429 complete records for all variables used in the revised analyses; therefore, a complete-case approach was applied and no imputation was performed.

### 2.2 Data collection and candidate variables

Available data included demographic information, anthropometric measures, questionnaire-derived lifestyle factors, health-examination comorbidity indicators, and routine laboratory indices. All biochemical measurements used in the present analyses, including glucose and lipid variables, were based on routine fasting blood specimens collected as part of the health-examination protocol. The analytic dataset did not contain the exact fasting duration for each individual. Hypertension, diabetes, and dyslipidemia were treated as dichotomous comorbidity indicators derived from examination-based indicators obtained during the routine health examination rather than from medication records. No medication-related variables were available in the analytic dataset; specifically, separate medication-class variables, including statins, antihypertensive agents such as ACE inhibitors or angiotensin receptor blockers, glucose-lowering drugs, and anti-inflammatory medications, were not available. Variables considered as covariates were selected *a priori* on clinical grounds and based on previous kidney epidemiology literature.

### 2.3 Exposure definition

CTI was the primary exposure. Because triglycerides and fasting plasma glucose were measured in mmol/L, values were converted to mg/dL before calculating TyG (triglycerides ×88.57 and fasting plasma glucose ×18.00). The TyG index was then calculated as ln [TG (mg/dL) × FPG (mg/dL)/2], a formula originally proposed and later validated as a surrogate marker of insulin resistance (15). CTI was derived as TyG + 0.412 × ln [hs-CRP (mg/L)], consistent with the published formulation of this index (26). For comparative analyses, remnant cholesterol (RC) was calculated as total cholesterol − LDL-C − HDL-C (33).

### 2.4 Outcome definition

The primary outcome was single-occasion reduced eGFR. Estimated glomerular filtration rate was calculated from serum creatinine using the 2021 CKD-EPI creatinine equation without race (34). Because this study relied on a single health examination rather than repeat measurements over at least 3 months, the main renal end point was defined as eGFR <90 mL/min/1.73 m² to capture early filtration reduction in a screening setting; it was not labeled or interpreted as CKD. We additionally reported the frequency of eGFR <60 mL/min/1.73 m², evaluated eGFR <75 mL/min/1.73 m² as a stricter threshold when event counts permitted, and examined albuminuria (urinary albumin-creatinine ratio ≥30 mg/g), positive urine protein on dipstick, and a combined phenotype of reduced eGFR or albuminuria.

### 2.5 Statistical analysis

Continuous variables are presented as mean (SD), and categorical variables as number (percentage). Baseline characteristics were compared across CTI quartiles using one-way analysis of variance, the Kruskal-Wallis test, or the χ² test, as appropriate. Multivariable logistic regression was used to estimate odds ratios (ORs) and 95% CIs for single-occasion reduced eGFR. Model 1 adjusted for age and sex. Model 2 further adjusted for body mass index (BMI), smoking, drinking, physical activity, and educational attainment. Model 3 additionally adjusted for hypertension, diabetes, dyslipidemia, and uric acid. We did not adjust for serum creatinine, blood urea nitrogen, ACR, or urine protein in the main model because these variables are either directly involved in outcome construction or may lie on the downstream pathway of renal injury. Linear trend across quartiles was assessed by assigning the ordinal quartile number as a continuous term.

Potential nonlinearity was examined using restricted cubic splines with four knots, following standard regression spline methodology (35). Discriminative performance was evaluated by ROC analysis, and correlated AUCs were compared using the DeLong method (36). Variance inflation factors (VIFs) were calculated for the fully adjusted model to assess multicollinearity among CTI and metabolic covariates. Prespecified subgroup analyses were performed according to age (<60 vs ≥60 years), sex, BMI (<24 vs ≥24 kg/m²), hypertension, diabetes, and dyslipidemia. Because the prevalence of single-occasion reduced eGFR was not rare, we additionally fitted a modified Poisson model with robust variance to estimate prevalence ratios (37). To complement the binary-outcome analysis, we also modeled continuous eGFR using multivariable linear regression. Additional sensitivity analyses excluded participants with hs-CRP >10 mg/L, excluded participants with diabetes, excluded those with hypertension or diabetes, excluded those with positive urine protein, replaced BMI with waist circumference in the fully adjusted model, and evaluated stricter or combined renal phenotypes. All analyses were two-sided, with P<.05 indicating statistical significance, and were performed using Python 3.11.

## 3 Results

### 3.1 Participant characteristics

The analytic sample included 1429 adults (mean [SD] age, 48.77 [11.28] years; 46.8% women). The mean (SD) eGFR was 97.46 (12.39) mL/min/1.73 m², and 398 participants (27.9%) met the prespecified definition of single-occasion reduced eGFR. Only 1 participant (0.1%) had eGFR <60 mL/min/1.73 m², whereas 55 participants (3.8%) had eGFR <75 mL/min/1.73 m². Across increasing CTI quartiles, participants were progressively older, more often male, had higher BMI, waist circumference, fasting glucose, triglycerides, uric acid, and serum creatinine, and had lower HDL-C and lower eGFR (all P<0.001 for most comparisons) (**Table 1**). The prevalence of reduced eGFR increased markedly across CTI quartiles, from 12.8% in Q1 to 20.4% in Q2, 32.5% in Q3, and 45.7% in Q4 (P<0.001).

**Table 1:** Baseline characteristics according to CTI quartiles.

Variable	Total	Q1	Q2	Q3	Q4	P value
Age, years	48.77 (11.28)	44.52 (10.80)	46.70 (11.00)	51.03 (10.48)	52.86 (10.87)	<0.001
Body mass index, kg/m²	24.25 (3.03)	22.17 (2.58)	23.58 (2.56)	24.71 (2.47)	26.55 (2.71)	<0.001
Waist circumference, cm	83.63 (7.65)	78.81 (7.00)	82.37 (6.48)	85.05 (6.86)	88.29 (6.90)	<0.001
Serum creatinine, μmol/L	76.27 (11.98)	70.54 (11.21)	74.75 (11.40)	78.06 (11.53)	81.74 (10.87)	<0.001
Blood urea nitrogen, mmol/L	4.73 (0.89)	4.39 (0.80)	4.61 (0.81)	4.85 (0.84)	5.08 (0.96)	<0.001
Uric acid, μmol/L	310.57 (60.53)	281.54 (54.41)	302.56 (55.91)	316.12 (56.47)	342.16 (58.88)	<0.001
Fasting plasma glucose, mmol/L	5.21 (1.00)	4.48 (0.39)	4.87 (0.55)	5.32 (0.71)	6.19 (1.20)	<0.001
Total cholesterol, mmol/L	4.45 (0.42)	4.29 (0.39)	4.43 (0.39)	4.48 (0.43)	4.61 (0.41)	<0.001
HDL-C, mmol/L	1.34 (0.21)	1.50 (0.17)	1.40 (0.15)	1.31 (0.17)	1.16 (0.18)	<0.001
LDL-C, mmol/L	2.72 (0.39)	2.52 (0.36)	2.66 (0.35)	2.76 (0.37)	2.93 (0.36)	<0.001
TyG index	8.58 (0.58)	7.93 (0.31)	8.40 (0.26)	8.73 (0.26)	9.26 (0.39)	<0.001
CTI	8.52 (0.74)	7.59 (0.31)	8.25 (0.15)	8.75 (0.14)	9.47 (0.39)	<0.001
Remnant cholesterol, mmol/L	0.40 (0.18)	0.27 (0.15)	0.37 (0.16)	0.41 (0.17)	0.53 (0.16)	<0.001
eGFR, mL/min/1.73 m²	97.46 (12.39)	103.35 (11.33)	100.13 (11.78)	94.94 (11.09)	91.38 (11.81)	<0.001
Triglycerides, mmol/L	1.45 (0.70)	0.81 (0.23)	1.18 (0.28)	1.52 (0.37)	2.28 (0.74)	<0.001
hs-CRP, mg/L	1.16 (1.07)	0.53 (0.34)	0.83 (0.46)	1.26 (0.96)	2.04 (1.44)	<0.001
Urinary albumin-to-creatinine ratio, mg/g	15.85 (20.82)	9.29 (10.94)	11.59 (11.68)	16.91 (18.46)	25.62 (31.34)	<0.001
Sex						<0.001
Female	669 (46.8%)	226 (63.1%)	175 (49.0%)	150 (42.0%)	118 (33.1%)	
Male	760 (53.2%)	132 (36.9%)	182 (51.0%)	207 (58.0%)	239 (66.9%)	
Urine protein						<0.001
Negative	1181 (82.6%)	337 (94.1%)	327 (91.6%)	283 (79.3%)	234 (65.5%)	
Trace	218 (15.3%)	20 (5.6%)	28 (7.8%)	65 (18.2%)	105 (29.4%)	
1+	30 (2.1%)	1 (0.3%)	2 (0.6%)	9 (2.5%)	18 (5.0%)	
Current smoking						<0.001
No	1055 (73.8%)	301 (84.1%)	258 (72.3%)	253 (70.9%)	243 (68.1%)	
Yes	374 (26.2%)	57 (15.9%)	99 (27.7%)	104 (29.1%)	114 (31.9%)	
Current drinking						<0.001
No	932 (65.2%)	266 (74.3%)	246 (68.9%)	230 (64.4%)	190 (53.2%)	
Yes	497 (34.8%)	92 (25.7%)	111 (31.1%)	127 (35.6%)	167 (46.8%)	
Physical activity						<0.001
Low	343 (24.0%)	66 (18.4%)	74 (20.7%)	89 (24.9%)	114 (31.9%)	
Moderate	722 (50.5%)	162 (45.3%)	194 (54.3%)	190 (53.2%)	176 (49.3%)	
High	364 (25.5%)	130 (36.3%)	89 (24.9%)	78 (21.8%)	67 (18.8%)	
Educational attainment						0.002
Middle school or below	393 (27.5%)	77 (21.5%)	87 (24.4%)	123 (34.5%)	106 (29.7%)	
High school/technical school	487 (34.1%)	118 (33.0%)	121 (33.9%)	119 (33.3%)	129 (36.1%)	
College	370 (25.9%)	108 (30.2%)	106 (29.7%)	72 (20.2%)	84 (23.5%)	
Postgraduate	179 (12.5%)	55 (15.4%)	43 (12.0%)	43 (12.0%)	38 (10.6%)	
Hypertension						<0.001
No	1118 (78.2%)	317 (88.5%)	290 (81.2%)	280 (78.4%)	231 (64.7%)	
Yes	311 (21.8%)	41 (11.5%)	67 (18.8%)	77 (21.6%)	126 (35.3%)	
Diabetes						<0.001
No	1309 (91.6%)	358 (100.0%)	351 (98.3%)	334 (93.6%)	266 (74.5%)	
Yes	120 (8.4%)	0 (0.0%)	6 (1.7%)	23 (6.4%)	91 (25.5%)	
Dyslipidemia						<0.001
No	856 (59.9%)	328 (91.6%)	290 (81.2%)	193 (54.1%)	45 (12.6%)	
Yes	573 (40.1%)	30 (8.4%)	67 (18.8%)	164 (45.9%)	312 (87.4%)	
Reduced eGFR						<0.001
No	1031 (72.1%)	312 (87.2%)	284 (79.6%)	241 (67.5%)	194 (54.3%)	
Yes	398 (27.9%)	46 (12.8%)	73 (20.4%)	116 (32.5%)	163 (45.7%)	

Values are presented as mean (SD) or n (%). P values were derived from one-way ANOVA or the χ² test as appropriate. CTI quartiles were defined as Q1 ≤7.984; Q2 7.985-8.505; Q3 8.506-9.003; and Q4 >9.003.

### 3.2 Association between CTI and reduced eGFR

In the primary logistic regression analysis, higher CTI was independently associated with single-occasion reduced eGFR (**Table 2**). In the fully adjusted model, each 1-SD increase in CTI was associated with a 45% increase in the odds of reduced eGFR (OR, 1.45; 95% CI, 1.11-1.90; P = 0.007). When CTI was modeled categorically, the highest quartile remained independently associated with reduced eGFR compared with the lowest quartile (OR, 2.11; 95% CI, 1.07-4.17; P = 0.031). The intermediate quartiles showed attenuated estimates after multivariable adjustment, consistent with substantial confounding by age, adiposity, and metabolic comorbidity. Accordingly, the fully adjusted linear trend across quartiles was borderline (P for trend=0.056), despite the statistically significant Q4-versus-Q1 contrast. Multicollinearity diagnostics did not indicate problematic collinearity in the fully adjusted model. The VIF for CTI was 2.44, and all VIFs were below 2.5, including BMI (1.94), uric acid (2.05), dyslipidemia (1.63), and diabetes (1.22). Because diabetes, dyslipidemia, and adiposity are biologically related to the CTI components, these adjusted estimates should be interpreted as conservative estimates of the CTI signal after accounting for overlapping metabolic burden.

**Table 2:** Association of CTI with reduced eGFR.

Exposure	Model 1		Model 2		Model 3	
	OR (95% CI)	P value	OR (95% CI)	P value	OR (95% CI)	P value
Per 1-SD increase	1.48 (1.23-1.78)	<0.001	1.42 (1.13-1.78)	0.003	1.45 (1.11-1.90)	0.007
Q1	Reference					
Q2	1.50 (0.86-2.63)	0.152	1.45 (0.82-2.55)	0.203	1.41 (0.80-2.49)	0.236
Q3	1.40 (0.83-2.37)	0.21	1.29 (0.73-2.25)	0.378	1.34 (0.76-2.37)	0.318
Q4	2.53 (1.50-4.27)	<0.001	2.08 (1.14-3.82)	0.018	2.11 (1.07-4.17)	0.031
P for trend		<0.001		0.032		0.056

Model 1 adjusted for age and sex. Model 2 additionally adjusted for BMI, smoking, drinking, physical activity, and educational attainment. Model 3 additionally adjusted for hypertension, diabetes, dyslipidemia, and uric acid. P values were obtained from Wald tests, and P for trend was assessed by modeling CTI quartiles as an ordinal variable. Reduced eGFR was defined as eGFR <90 mL/min/1.73 m².

### 3.3 Dose-response and discriminative performance

**Figure 1:**
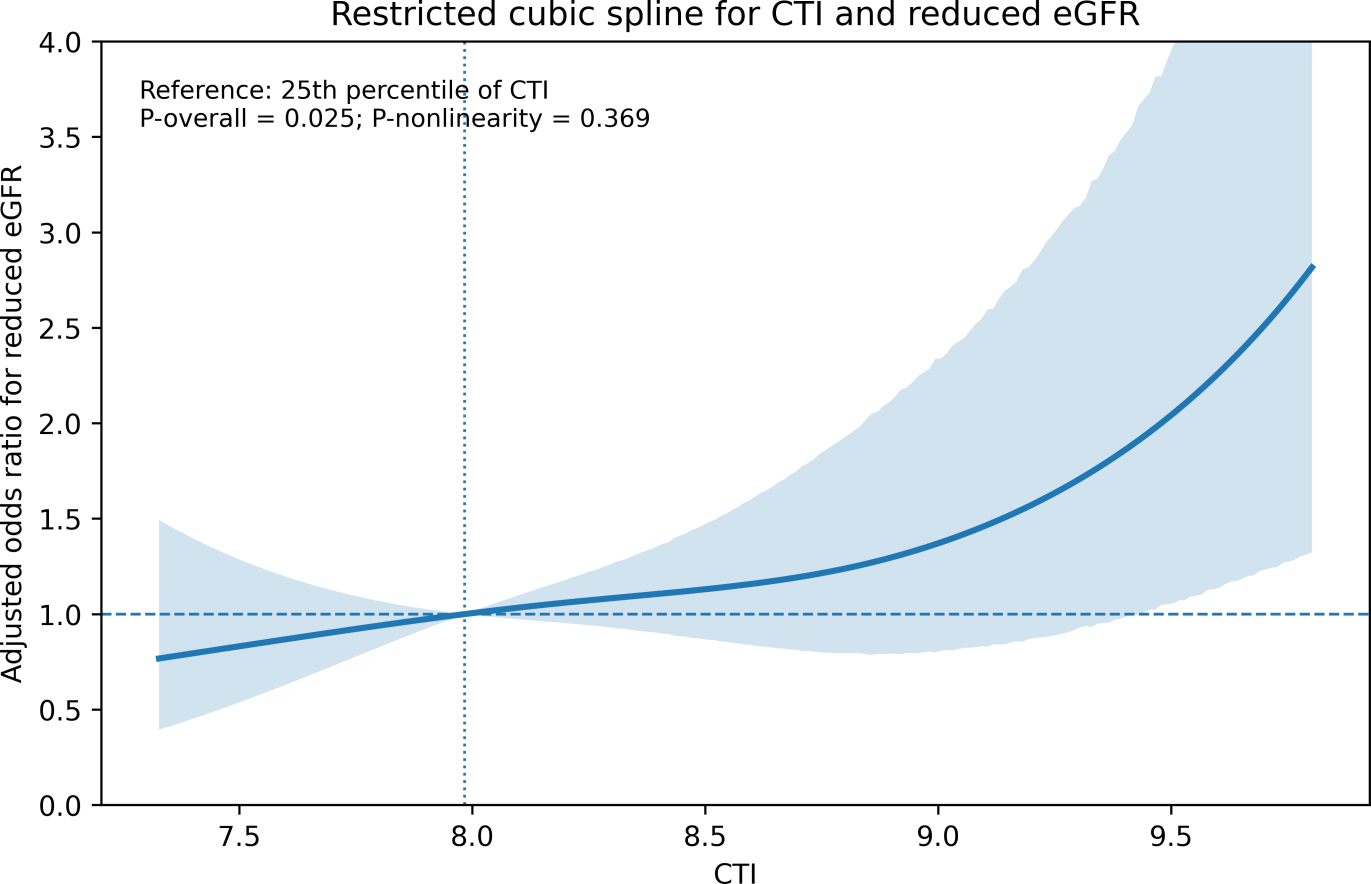
Restricted cubic spline for CTI and reduced eGFR. Adjusted odds ratios are plotted relative to the 25th percentile of CTI. The spline model was adjusted for age, sex, BMI, smoking, drinking, physical activity, educational attainment, hypertension, diabetes, dyslipidemia, and uric acid. Shaded areas indicate 95% CIs. The plotted range is shown between the 5th and 95th percentiles to improve visual stability at the extremes.

Restricted cubic spline analysis demonstrated a statistically significant overall association between CTI and reduced eGFR (P-overall=0.025), whereas the nonlinearity test was not significant (P-nonlinearity=0.369), indicating an approximately linear positive dose-response pattern over the observed range (**Figure 1**). In the fully adjusted linear regression model, each 1-SD increase in CTI was associated with a lower eGFR (β, -1.29 mL/min/1.73 m²; 95% CI, -1.78 to -0.81; P<0.001), supporting concordance between the binary and continuous outcome analyses.

**Figure 2:**
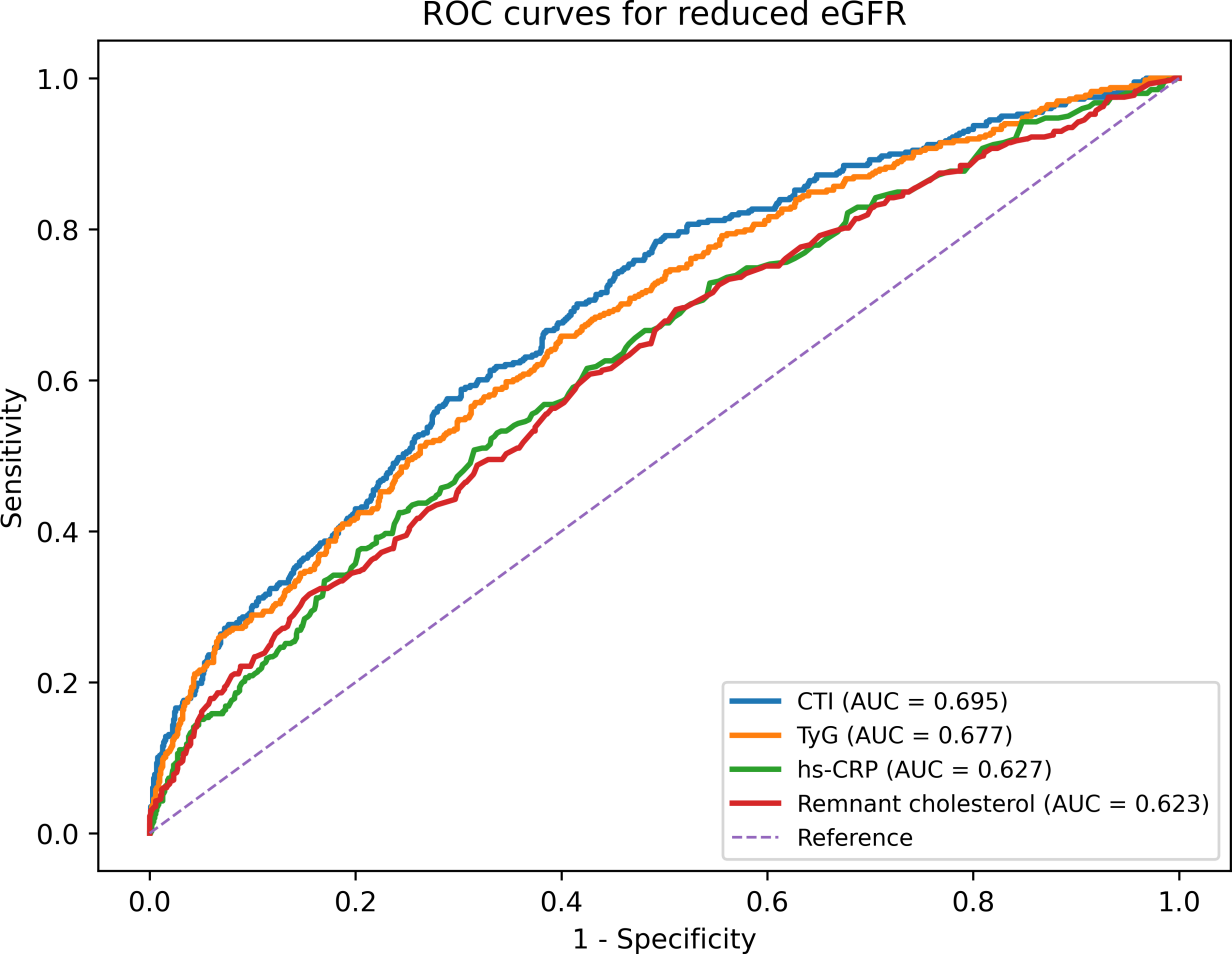
Receiver operating characteristic curves for CTI, TyG, hs-CRP, and remnant cholesterol. CTI showed modest but statistically superior discrimination for reduced eGFR relative to the comparator biomarkers; the absolute AUC remained limited and should not be interpreted as sufficient for stand-alone screening.

ROC analyses showed that CTI had modest discrimination for reduced eGFR, with an AUC of 0.695 (95% CI, 0.662-0.725), which was significantly greater than the AUCs for TyG (0.677; 95% CI, 0.646-0.708; P = 0.021 vs CTI), hs-CRP (0.627; 95% CI, 0.597-0.659; P<0.001 vs CTI), and remnant cholesterol (0.623; 95% CI, 0.590-0.653; P<0.001 vs CTI) (**Figure 2**, **Table 3**). These findings suggest that integrating inflammatory and metabolic information modestly improves discrimination relative to the individual component markers or a related lipid-derived marker. However, the absolute discriminatory performance remained limited and should not be interpreted as sufficient for stand-alone clinical screening.

**Table 3:** ROC comparison for reduced eGFR.

Marker	AUC (95% CI)	Comparison with CTI (P value)
CTI	0.695 (0.662-0.725)	Reference
TyG	0.677 (0.646-0.708)	0.021
hs-CRP	0.627 (0.597-0.659)	<0.001
RC	0.623 (0.590-0.653)	<0.001

AUCs were compared with CTI using the DeLong method.

### 3.4 Subgroup and sensitivity analyses

**Figure 3:**
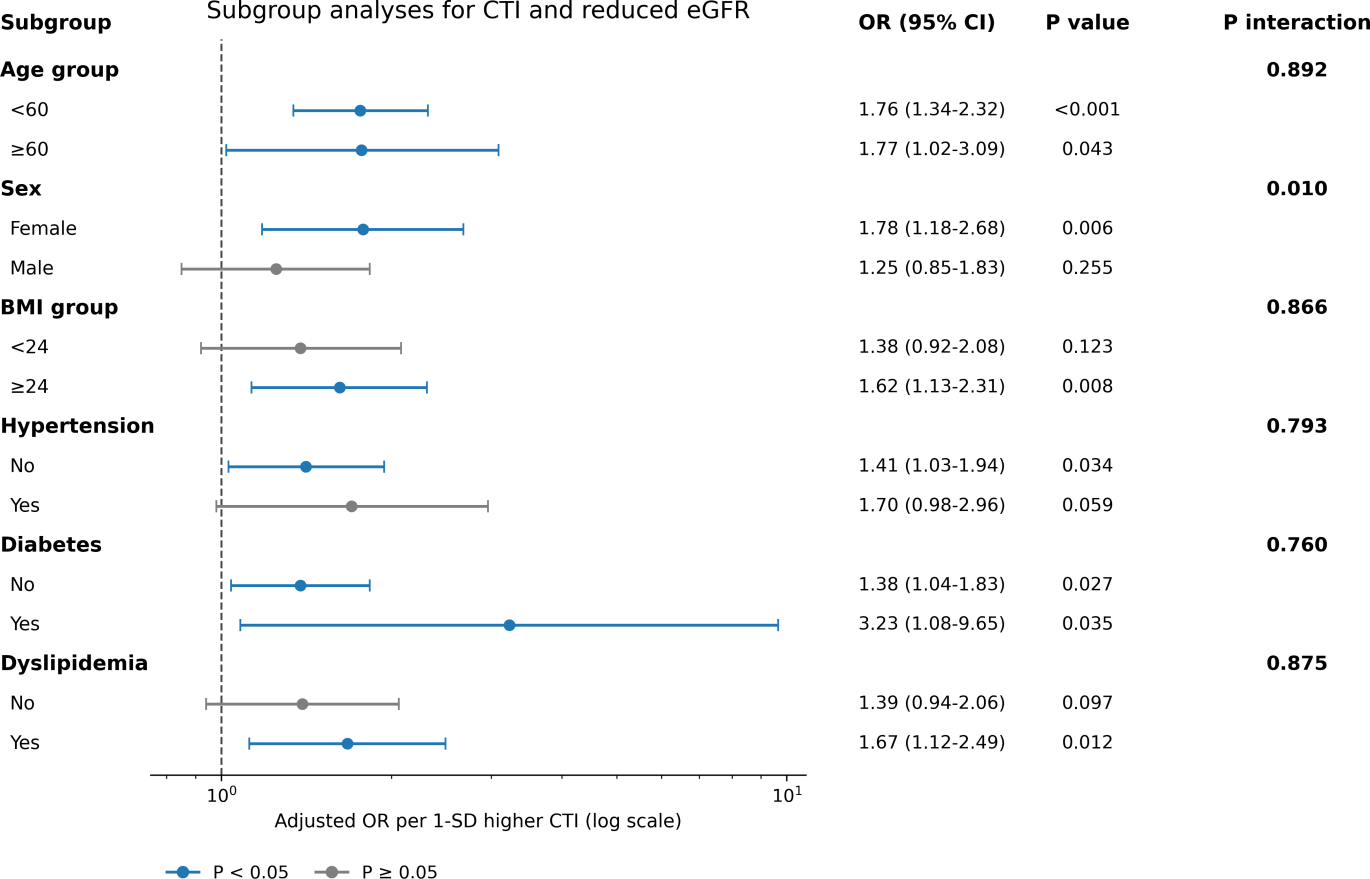
Subgroup analyses for the association between CTI and reduced eGFR. Points represent adjusted odds ratios per 1-SD higher CTI and horizontal bars represent 95% CIs. Interaction P values are shown for each subgroup variable. The figure was regenerated using only two colors to distinguish subgroup estimates with P<0.05 from those with P≥0.05, with larger labels to improve readability.

In subgroup analyses, the association between CTI and reduced eGFR was generally directionally consistent across strata (**Figure 3**, **Table 4**). A statistically significant interaction was observed for sex (P-interaction=0.010), with a stronger association in women (OR per 1-SD, 1.78; 95% CI, 1.18-2.68) than in men (OR, 1.25; 95% CI, 0.85-1.83). No significant interaction was detected for age group, BMI category, hypertension, diabetes, or dyslipidemia. Notably, the point estimate was numerically larger in participants with diabetes (OR, 3.23; 95% CI, 1.08-9.65), although the interaction test was not significant and the subgroup was relatively small.

**Table 4:** Subgroup analyses for the association between CTI and reduced eGFR.

Subgroup	OR (95% CI)	P value	P for interaction
Age group			
<60	1.76 (1.34-2.32)	<0.001	0.892
≥60	1.77 (1.02-3.09)	0.043	
Sex			
Female	1.78 (1.18-2.68)	0.006	0.01
Male	1.25 (0.85-1.83)	0.255	
BMI group			
<24	1.38 (0.92-2.08)	0.123	0.866
≥24	1.62 (1.13-2.31)	0.008	
Hypertension			
No	1.41 (1.03-1.94)	0.034	0.793
Yes	1.70 (0.98-2.96)	0.059	
Diabetes			
No	1.38 (1.04-1.83)	0.027	0.76
Yes	3.23 (1.08-9.65)	0.035	
Dyslipidemia			
No	1.39 (0.94-2.06)	0.097	0.875
Yes	1.67 (1.12-2.49)	0.012	

Effect estimates are adjusted ORs per 1-SD higher CTI, with covariates identical to the fully adjusted model except for the stratifying variable.

Sensitivity analyses supported the robustness of the main findings while also illustrating the limited number of more advanced renal-function events (**Table 5**). The fully adjusted association remained significant after excluding participants with hs-CRP >10 mg/L (OR, 1.46; 95% CI, 1.11-1.92), after excluding participants with diabetes (OR, 1.38; 95% CI, 1.04-1.83), and after replacing BMI with waist circumference in the fully adjusted model (OR, 1.47; 95% CI, 1.14-1.91). When a modified Poisson model with robust variance was used, the association remained significant (prevalence ratio, 1.19; 95% CI, 1.06-1.32; P = 0.002). For the stricter eGFR <75 mL/min/1.73 m² threshold, the association was directionally similar but borderline (OR, 1.75; 95% CI, 0.99-3.10; P = 0.056). Because only 1 participant had eGFR <60 mL/min/1.73 m², multivariable analysis using this threshold was not performed. The point estimate was attenuated when analyses were restricted to participants without hypertension or diabetes and when those with positive urine protein were excluded, but the direction of association remained unchanged.

**Table 5:** Sensitivity and secondary outcome analyses.

Analysis	N	Events	Effect estimate (95% CI)	P value
Exclude hs-CRP >10 mg/L	1428	398	1.46 (1.11-1.92)	0.006
Exclude diabetes	1309	322	1.38 (1.04-1.83)	0.027
Exclude hypertension or diabetes	1045	192	1.31 (0.93-1.83)	0.118
Exclude positive urine protein	1181	294	1.28 (0.93-1.74)	0.125
Replace BMI with waist circumference	1429	398	1.47 (1.14-1.91)	0.003
eGFR <75 mL/min/1.73 m²	1429	55	1.75 (0.99-3.10)	0.056
Combined reduced eGFR or albuminuria	1429	507	1.81 (1.45-2.27)	<0.001
Albuminuria (ACR ≥30 mg/g)	1429	190	2.50 (1.90-3.29)	<0.001
Positive urine protein	1429	248	2.39 (1.87-3.06)	<0.001
Modified Poisson model (prevalence ratio)	1429	398	1.19 (1.06-1.32)	0.002
Linear regression for continuous eGFR, β (95% CI)	1429	–	-1.29 (-1.78 to -0.81)	<0.001

Albuminuria was defined as ACR ≥30 mg/g. Positive urine protein was defined as trace or higher on urine dipstick category. Reduced eGFR was defined as eGFR <90 mL/min/1.73 m² unless otherwise specified. Only 1 participant (0.1%) had eGFR <60 mL/min/1.73 m²; therefore, multivariable analysis using this threshold was not performed. Modified Poisson models are presented as prevalence ratios.

To examine consistency across renal phenotypes, we further analyzed albuminuria, positive urine protein, and a combined phenotype of reduced eGFR or albuminuria. In the fully adjusted model, each 1-SD increase in CTI was associated with substantially higher odds of albuminuria (OR, 2.50; 95% CI, 1.90-3.29; P<0.001), positive urine protein (OR, 2.39; 95% CI, 1.87-3.06; P<0.001), and the combined reduced eGFR-or-albuminuria phenotype (OR, 1.81; 95% CI, 1.45-2.27; P<0.001). These concordant associations with functional and urinary phenotypes support the internal consistency of the findings, although they remain cross-sectional associations.

## 4 Discussion

In this hospital-based cross-sectional study of adults undergoing routine health examinations, higher CTI was independently associated with a higher prevalence of single-occasion reduced eGFR. The association persisted after extensive adjustment, remained directionally consistent in sensitivity analyses, and was also evident for albuminuria, positive urine protein, and the combined reduced eGFR-or-albuminuria phenotype. However, the primary endpoint largely represented mild filtration reduction: only 1 participant had eGFR <60 mL/min/1.73 m². These results should therefore be interpreted as evidence of association with early or mild renal-function reduction rather than as evidence of established CKD or future renal decline.

These findings add to the emerging literature on CTI by focusing on a routine health examination population, a setting distinct from studies centered on clinically recognized CKD or broader population datasets. The incremental contribution of the present study is not to establish a new causal mechanism, but to show that the CTI signal is detectable in an asymptomatic screening context, to compare CTI with TyG, hs-CRP, and remnant cholesterol, to evaluate urinary phenotypes and a stricter eGFR threshold, and to address multicollinearity and clinical interpretability. In this sense, our findings are confirmatory of the general CTI-kidney association but extend it to an early-detection setting with additional robustness checks.

Several biological pathways may plausibly underlie or accompany this association, while acknowledging that the cross-sectional design cannot determine directionality. The TyG component reflects insulin-resistant metabolism, which has been linked to glomerular hyperfiltration, renin-angiotensin system activation, lipid deposition, oxidative stress, and mitochondrial stress within renal tissue (5, 6, 16–20). Meanwhile, hs-CRP captures low-grade systemic inflammation, a process closely linked to endothelial dysfunction, tubular injury, and loss of renal reserve (7–12, 22–25). Conversely, reduced kidney function itself may intensify inflammation and alter lipid and glucose metabolism, making reverse causation biologically plausible.

An important observation in our analysis is that CTI performed better than either TyG or hs-CRP alone, but this improvement should be interpreted conservatively. The AUC improvement was statistically significant, yet the absolute AUC remained below 0.70. Moreover, we did not test calibration, net reclassification, decision-curve benefit, external validation, or incremental value beyond established renal risk assessment approaches. Therefore, CTI should be considered a simple adjunctive marker for research or preliminary risk stratification rather than a replacement for standard renal evaluation.

The shape of the dose-response relationship also deserves attention. We did not observe convincing statistical evidence of marked nonlinearity; instead, the odds of reduced eGFR increased generally across the observed CTI distribution. This finding differs somewhat from reports in other disease settings that suggested possible threshold behavior (31). In a relatively healthy examination cohort, the association may reflect a gradual cross-sectional accumulation of metabolic-inflammatory burden rather than a clearly separable cutoff.

The stronger association observed in women should be interpreted carefully but is worthy of further study. Potential explanations include sex differences in fat distribution, inflammatory tone, postmenopausal metabolic change, endothelial susceptibility, or differences in creatinine generation that may influence creatinine-based eGFR estimation. Women in health examination settings may also differ from men in health-seeking behavior and comorbidity detection. Because this was a single-center cross-sectional analysis and prior CTI-related kidney analyses have not shown entirely uniform sex patterns (31), the sex interaction should be viewed as hypothesis-generating and requires replication.

From a clinical perspective, CTI is appealing because it is simple to calculate and relies on laboratory tests already obtained in many health examination programs. Nevertheless, its clinical role should be limited to an adjunctive signal of metabolic-inflammatory burden. It is not a diagnostic substitute for repeat eGFR testing, albuminuria assessment, medication review, or formal CKD risk evaluation. The modest ROC performance suggests that CTI alone should not be used to classify individuals as having kidney disease, but it may help prompt closer review of conventional renal and cardiometabolic risk factors when interpreted in context.

This study has several limitations. Its cross-sectional design precludes inference regarding temporality, causality, or prediction, and reverse causation is possible because impaired renal function itself may aggravate metabolic and inflammatory abnormalities. The data were derived from a single tertiary hospital health examination center, which may limit generalizability. Residual confounding cannot be excluded, particularly for dietary factors, socioeconomic factors beyond education, unmeasured comorbidity burden, and medication exposure. Medication-related information was not available in the analytic dataset; therefore, we could not account for statins, ACE inhibitors or angiotensin receptor blockers, glucose-lowering agents, anti-inflammatory medications, or other drug classes that may influence inflammation, glucose metabolism, lipid profiles, or renal function. Although blood samples were collected under routine fasting conditions, exact individual fasting duration was not recorded. In addition, reduced eGFR was defined from a single creatinine measurement; therefore, our findings should not be interpreted as evidence of CKD without chronicity being established (3). The very low number of participants with eGFR <60 mL/min/1.73 m² also limited our ability to evaluate clinically more advanced renal impairment. Prospective, multicenter studies with repeated kidney measurements, medication information, and external validation are needed.

## 5 Conclusions

In conclusion, higher CTI was independently associated with a higher prevalence of single-occasion reduced eGFR in adults undergoing routine health examinations. The relation was broadly linear, directionally robust across sensitivity analyses, and accompanied by associations with albuminuria and positive urine protein. Given the cross-sectional design and modest discrimination, CTI should be viewed as an adjunctive marker of metabolic-inflammatory burden rather than a diagnostic or predictive tool. Prospective studies with repeated renal measurements are needed to determine whether CTI predicts subsequent renal function decline.

## Data Availability

The original contributions presented in the study are included in the article/supplementary material. Further inquiries can be directed to the corresponding author.
